# Osteoid osteoma near the intervertebral foramen may induce radiculopathy through tumorous inflammation

**DOI:** 10.1186/1746-1596-6-10

**Published:** 2011-01-19

**Authors:** Michihisa Zenmyo, Takuya Yamamoto, Yasuhiro Ishidou, Setsuro Komiya, Kosei Ijiri

**Affiliations:** 1 Orthopaedic Surgery, Graduate School of Medical and Dental Sciences, Kagoshima University, Kagoshima, Japan

## Abstract

Osteoid osteoma of the spine is a relatively rare bone-forming tumor. Pain that is worse at night and relieved by aspirin and muscle contracture are the most characteristic symptoms of spinal osteoid osteoma. Although radicular pain occasionally occurs in spinal osteoid osteoma, spinal cord and nerve root compression is absent in most cases. Although radicular pain appears to be associated with tumorous inflammation, there have been no presentations of histological findings of inflammation around the nerve root. We present here two rare cases of spinal osteoid osteoma causing radiculopathy and the first histological evidence of tumorous inflammation as a cause of radiculopathy in osteoid osteoma near the intervertebral foramen.

## Background

Osteoid osteoma is a benign bone tumor typically affecting the long bones in young patients. It is small but painful, and has a central nidus that rarely exceeds 1.5 cm. Vertebral localization is uncommon (about 10%), and when it occurs in the spine lumbar lesions are most common (59%). The most common presenting symptom is pain that is worse at night and relieved by aspirin. Greco et al. reported that large amounts of prostaglandin E2 and prostacyclin released from the nidus are related to the sclerosis and pain in osteoid osteoma [1].

Although there have been several reports on osteoid osteoma, only six cases of osteoid osteoma with radiculopathy have been reported in the literature [2-4]. In most such cases, radiculopathy appeared to have been induced by nerve compression, and the cause of radiculopathy without nerve compression in patients with osteoid osteoma was not discussed. We report here rare cases of osteoid osteoma in the sacral lamina and the thoracic facet causing radiculopathy. We present histological findings for the soft tissue around the affected nerve root, and suggest for the first time that radiculopathy with osteoid osteoma may be induced by tumorous inflammation.

## Case Presentation

### Case 1

The patient was a 15-year-old boy without medical or family history of neoplastic disease. Low back pain and numbness in the posterior region of the left thigh were noted. Thereafter, the pain and numbness became worse at night. The pain was controlled with nonsteroidal anti-inflammatory drugs (NSAIDs), with gradual interval shortening. Finally, the patient was hospitalized in the author's department for progression of signs and symptoms.

On admission, he presented with severe limitation of lumbar spinal range of motion. He reported strong sciatic pain in his buttocks and left thigh at night. However, neurological examination was normal. The straight leg-raising test yielded increasing buttock pain on the left side.

**Figure 1:**
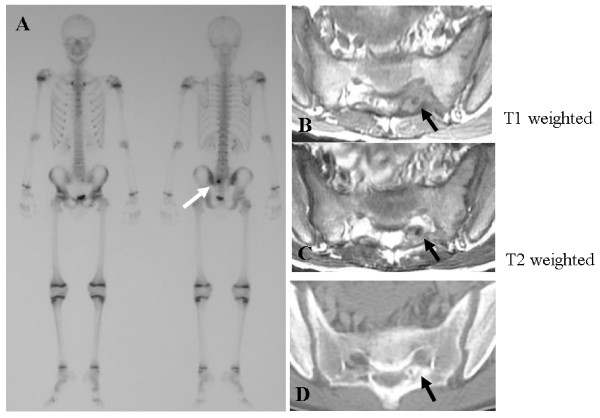
**Technetium bone scan, MRI and CT**. A; Technetium bone scan was useful in localizing the lesion. B, C; MRI revealed an intramedullary lesion, with isointense low signal on T1-weighted images and isointense high signal on T2-weighted images. D; Intense reactive sclerosis surrounding the nidus was seen on CT images.

Conventional radiographic examination revealed few distinct abnormalities in the lumbosacral spine. Radionuclide bone scan, which revealed increased uptake of isotope in the S1 lamina on the left side, was useful in localizing the lesion (Figure 1A). Magnetic resonance imaging (MRI) revealed an intramedullary lesion, with isointense low signal on T1-weighted images and isointense high signal on T2-weighted images (Figure 1B, C). The lesion was enhanced by gadolinium (Gd-DTPA). Computed tomography (CT) demonstrated intense reactive sclerosis surrounding the nidus (Figure 1D). A clinicoradiological diagnosis of osteoid osteoma was entertained.

The patient underwent surgical curettage of the lesion. Complete excision of the reactive sclerosis and the nidus was performed. The patient reported immediate relief of his pain just after surgery.

**Figure 2:**
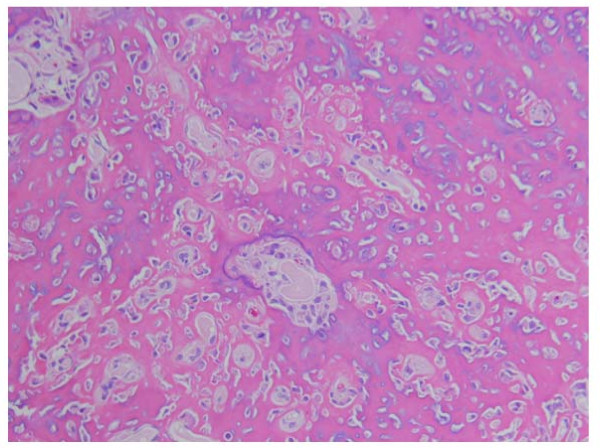
**Histological feature of the nidus (×40)**. H.E. staining of the lesion revealed that the nidus included anastomosing trabeculae of osteoid and woven bone.

On pathologic examination, the nidus tissue was well-circumscribed by reactive sclerosis. The nidus exhibited anastomosing bony trabeculae of various widths, and the bony trabeculae were rimmed by a single layer of osteoblasts. The intertrabecular spaces exhibited fibrovascular proliferation. These findings yielded the pathologic diagnosis of osteoid osteoma (Figure 2).

### Case 2

A 34-year-old man presented with pain in the right side of the back radiating to the anterior chest for a year. The pain was predominantly nocturnal and partially relieved by NSAIDs. On consultation, the patient complained of marked pain, but no sensory abnormalities or muscle weakness was noted. No rectal disorder was observed.

**Figure 3:**
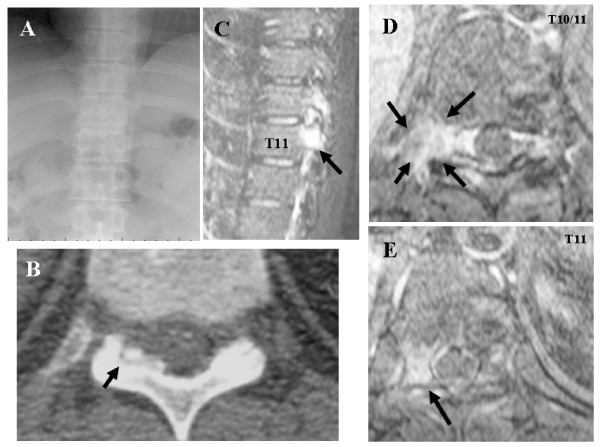
**Conventional roentgenogram, CT and Gd-enhanced MR images**. A; Anteroposterior radiograph of the thoracic spine revealed no abnormalities. B: CT revealed marked perilesional sclerosis of bone in the right facet. C, D, and E: Gd-enhanced area extended over the intervertebral foramen and the nerve root.

Anteroposterior radiographs of the thoracic spine revealed few distinct abnormalities in the thoracic spine (Figure 3A). Radionuclide bone scan revealed increased uptake of isotope in T11 on the right side. Although MRI demonstrated signal alteration in the facet of T10/11, no nidus was visualized. A high-intensity area on T2-weighted images that was enhanced by Gd-DTPA was present over the T10 nerve root (Figure 3C-E). These MRI findings indicated inflammation and consequent edema. In contrast to MRI, CT revealed intense reactive sclerosis surrounding the nidus in the tip of the superior articular process of T11 (Figure 3B), with no compression of the nerve root on axial view. Selective T10 nerve root block was performed as a diagnostic-therapeutic test, with improvement of the radiating pain.

**Figure 4:**
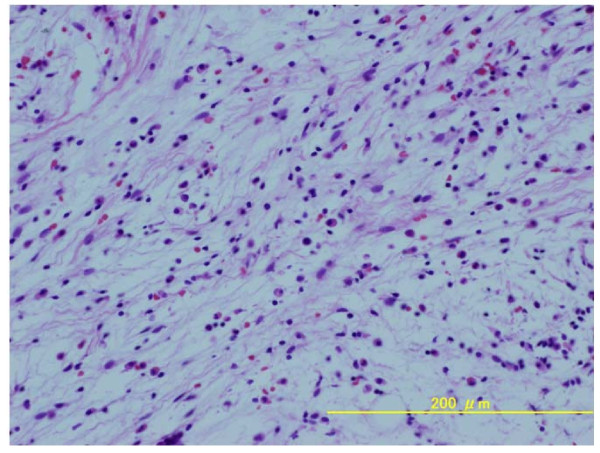
**Histological feature of the soft tissue around the nerve root**. Photomicrograph of the soft tissue around the nerve root showing infiltration by chronic inflammatory cells including lymphocytes and plasma cells.

Surgery with a posterior approach was performed. The nidus in the superior articular process was identified and "en bloc" resection was achieved. Some pieces of soft tissue around the facet joint and nerve root were taken for pathologic examination, which confirmed the diagnosis of osteoid osteoma and revealed the presence of chronic inflammation in the soft tissue around the nerve root (Figure 4).

## Discussion

Osteoid osteoma of the spine is a relatively rare bone tumor. It is reported that 10% of osteoid osteomas occur in the spine. Patients with osteoid osteoma usually present with complaints of local pain and/or swelling. Classically, the pain associated with this lesion is worse at night and partially relieved with NSAIDs. Although patients with spinal osteoid osteoma usually exhibit painful scoliosis, presentation with isolated root irritation is relatively rare. Although our first patient presented with typical local pain, the diagnosis was delayed because of the coexisting isolated root irritation. Our second patient underwent T10 nerve root block, which was effective for the radiculopathy but not local pain. Surgical resection relieved both radicular and local pain. These findings indicated that the radicular pain was induced by tumorous inflammation. We have presented here for the first time histological findings around the nerve root suggesting that chronic inflammation may induce radiculopathy. Large amounts of prostaglandin E2 and prostacyclin released from the nidus may be related to the isolated root irritation [1].

The diagnosis of osteoid osteoma of the spine is challenging, since conventional radiography is not useful for small lesions. MRI is useful not only for illustrating the lesion but also for demonstrating the existence of local inflammation and bone edema, especially on T2-weighted or Gd-enhanced images. In case 2, a high-intensity area including the intervertebral foramen and the nerve root were well-enhanced by Gd on T2-weighted images. In addition to MRI, radionuclide bone scanning was useful not only for localizing the lesion but also for revealing its intense activity, as described Schmitz et al. and Boretz et al [5,6]. We wish to emphasize the efficacy of radionuclide bone scanning as an aid to the early diagnosis of this painful lesion of the spine.

For surgical treatment, excision of the nidus has been reported to be effective in more than 95% of cases [7,8]. Complete resection of the nidus is a key to achieving remission of pain, with the assistance of CT imaging to localize the nidus preoperatively. Abe et al. advocated en bloc resection to facilitate precise operative procedures [9], as have other authors [10,11]. Rates of local recurrence have been found to be as high as 28% [12]. As described by Villas et al. [3], however, en bloc resection is not always appropriate in the spine. Recently, CT-guided radiofrequency ablation has been used to treat osteoid osteoma [13,14]. It has been proven to be an acceptable, safe, minimally invasive, and cost-effective treatment for this benign but painful tumor. Although we considered CT-guided radiofrequency ablation for the tumors in our patients, the S2 and T10 roots were very close to the nidus, and the possibility of injuring these nerve roots therefore existed. We therefore performed excision by curettage of the nidus and surrounding sclerotic bone, with complete remission of symptoms.

## Conclusions

We have described rare cases of osteoid osteoma in the spine that induced radiculopathy. We have presented the first histological evidence of tumorous inflammation causing radiculopathy in osteoid osteoma near the intervertebral foramen.

## Consent

Written informed consent was obtained from the patients for publication of this case report and any accompanying images. Copies of the written consents are available for review by the Editor-in-Chief of this journal.

## Competing interests

The authors declare that they have no competing interests.

## Authors' contributions

ZM and YI carried out the histochemical studies. TY and KI participated in the surgery and the design of the study, and KI contributed in the statistical analysis. SK and KI conceived the study and drafted the manuscript. All authors read and approved the final manuscript.
